# Supplementary material for: Complexome profiling of the Chlamydomonas psb28 mutant reveals TEF5 as an early PSII assembly factor
Source: Plant Cell. 2025 Mar 17;37(6):koaf055. doi: 10.1093/plcell/koaf055 (PMC12164590; doi:10.1093/plcell/koaf055)
Supplement: koaf055_Supplementary_Data [file koaf055_supplementary_data.zip › psb28_interactive.zip!/plots/intensity/qualityPlots/peptides/PRPL24_Cre16.g652550.t1.2.html]

### Cre16.g652550.t1.2

Trivial name: PRPL24  
SeqCoverage [median]: 12.90  
Mass: 18.27 kDa  
  
 

| Sequence | MissedCleavage | Mass [Da] | wtIntensity | muIntensity | Score |
| --- | --- | --- | --- | --- | --- |
| KGDTVQIIAGK | 1 | 1128.65 | 0.00 | 438.78 | 84.21 |
| VVVEGVNQGQK | 0 | 1155.62 | 510.47 | 1465.56 | 93.37 |
|  |  |  | 510.5 | 1904.3 |  |
